# Late-onset cobalamin C deficiency type in adult with cognitive and behavioral disturbances and significant cortical atrophy and cerebellar damage in the MRI: a case report

**DOI:** 10.3389/fneur.2023.1308289

**Published:** 2023-12-12

**Authors:** Miao Sun, Yingjie Dai

**Affiliations:** Department of Neurology, General Hospital of Northern Theater Command, Shenyang, China

**Keywords:** methylmalonic acidemia, bilateral cerebellar lesions, reversible DWI change, cblC, MMACHC

## Abstract

**Background:**

Late-onset cobalamin C (cblC) deficiency is associated with a wide range of neurological and psychiatric symptoms, hematological manifestations, anorexia, renal failure, ocular abnormalities, dermatitis, and pancreatitis. However, the neuroimaging characteristics of late-onset cblC deficiency remain insufficiently documented. Common findings include diffuse white matter swelling, varying degrees of severe leukoaraiosis, hydrocephalus, corpus callosum atrophy, and symmetric bilateral basal ganglia lesions. In this report, we present a case of late-onset cblC deficiency in adults presenting with cerebellar ataxia as the primary symptom. The MRI findings revealed bilateral lateral cerebellar hemispheres exhibiting symmetric hyperintensity, primarily observed in diffusion-weighted imaging (DWI), which is a rarely reported imaging change in this context.

**Case presentation:**

Our patient was a male who experienced symptoms starting at the age of 30 years, including unsteady walking, apparent cerebellar ataxia, and cognitive impairment upon nervous system examination. Brain magnetic resonance imaging (MRI) exhibited symmetric hyperintensity in the bilateral lateral cerebellar hemispheres, predominantly manifested in DWI, without any enhancement. Subsequently, significantly elevated blood total homocysteine and urinary methylmalonic acid levels were observed. Genetic analysis confirmed the presence of MMACHC compound heterozygous mutants c.482G > A and c.609G > A, thus confirming the diagnosis of cblC deficiency. These variants were classified as likely pathogenic following the guidelines of the American College of Medical Genetics and Genomics (ACMG) and were verified using Sanger sequencing. Following treatment, the patient experienced improvements in walking ability and cognition, a significant decrease in blood total homocysteine levels, and reversal of the imaging lesions.

**In conclusion:**

Late-onset cblC deficiency presents with diverse clinical and imaging manifestations. Early diagnosis and treatment are crucial in achieving a favorable prognosis. This case serves as a reminder to clinicians not to overlook genetic metabolic disorders, particularly those causing multisite damage, in adult patients with undiagnosed neurological disorders, especially those affecting the cerebellum. Notably, methylmalonic acidemia should be considered within the spectrum of bilateral cerebellar lesions.

## 1 Background

Methylmalonic acidemia (MMA) is a rare autosomal recessive genetic metabolic disorder characterized by defects in methylmalonyl-CoA mutase or cobalamin metabolism within the mitochondria. Consequently, methylmalonic acid, an intermediate product, accumulates in the body, resulting in multisystem damage. While primarily observed in infants and young children, CblC deficiency, a subtype of MMA, is less common in adults. Clinical and imaging manifestations of CblC deficiency exhibit significant heterogeneity (1). Infrequently, cerebellar ataxia manifests as the primary symptom, accompanied by bilateral lateral cerebellar hemispheres showing symmetric hyperintensity on DWI. This case report describes an adult patient with CblC deficiency presenting with bilateral cerebellar damage as the main clinical manifestation.

## 2 Case presentation

A 30-year-old male presented with a 7-day history of slow response and unsteady walking, as reported by his family. Clinical features included impaired calculation skills, limited language expression, unresponsiveness, difficulty climbing stairs, inability to walk in a straight line, and irritability. No other symptoms such as weakness, numbness, fever, cough, diarrhea, headache, nausea, vomiting, altered consciousness, or convulsions were observed. Skin manifestations, such as resembling staphylococcal scalded skin syndrome or a diffuse erythema with superficial erosions, desquamation, and cheilitis resembling acrodermatitis enteropathica, were not found in our patient. The patient had an unremarkable medical and developmental history, and his relatives did not exhibit similar symptoms. However, his scholastic performance was notably poor compared to his peers, having completed only the 8th grade. Neurological examination revealed delayed reaction times, impaired calculation ability, significant dysmetria on heel–knee–shin testing, and a positive Romberg test. Plasma total homocysteine 296.90 μmol/L (normal value 0.0 ~ 15.0 μmol/L) and D-dimer 9.38 ng/mL (normal value 0.0 ~ 0.55 ng/mL) are elevated.

**Figure 1:**
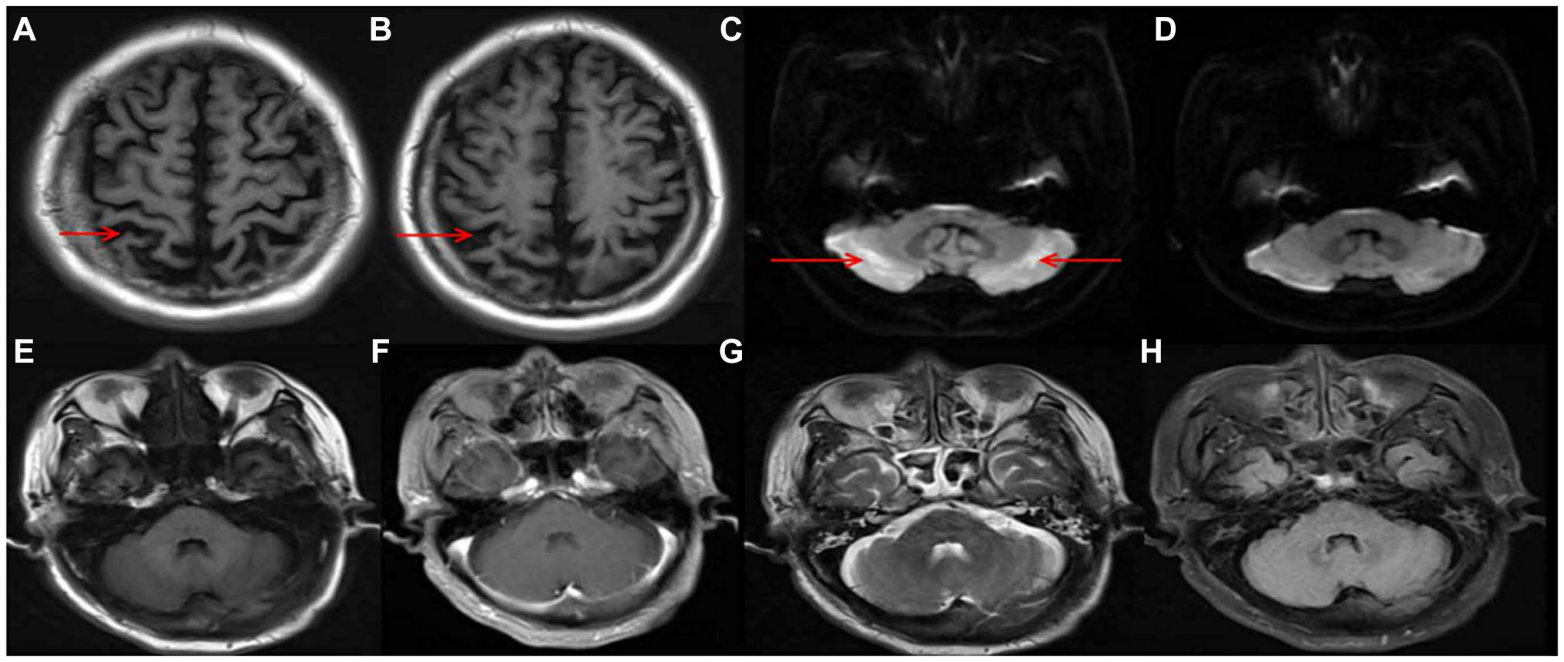
Cortical atrophy in T1 **(A,B)**; symmetric hyperintensity in the bilateral lateral cerebellar hemispheres, predominantly manifested in DWI **(C)**; reversal of the of cerebellar lesions after treatment **(D)**; T1/ enhancement/ T2/ FLAIR image no significant changes in cerebellar **(E–H)**.

**Figure 2:**
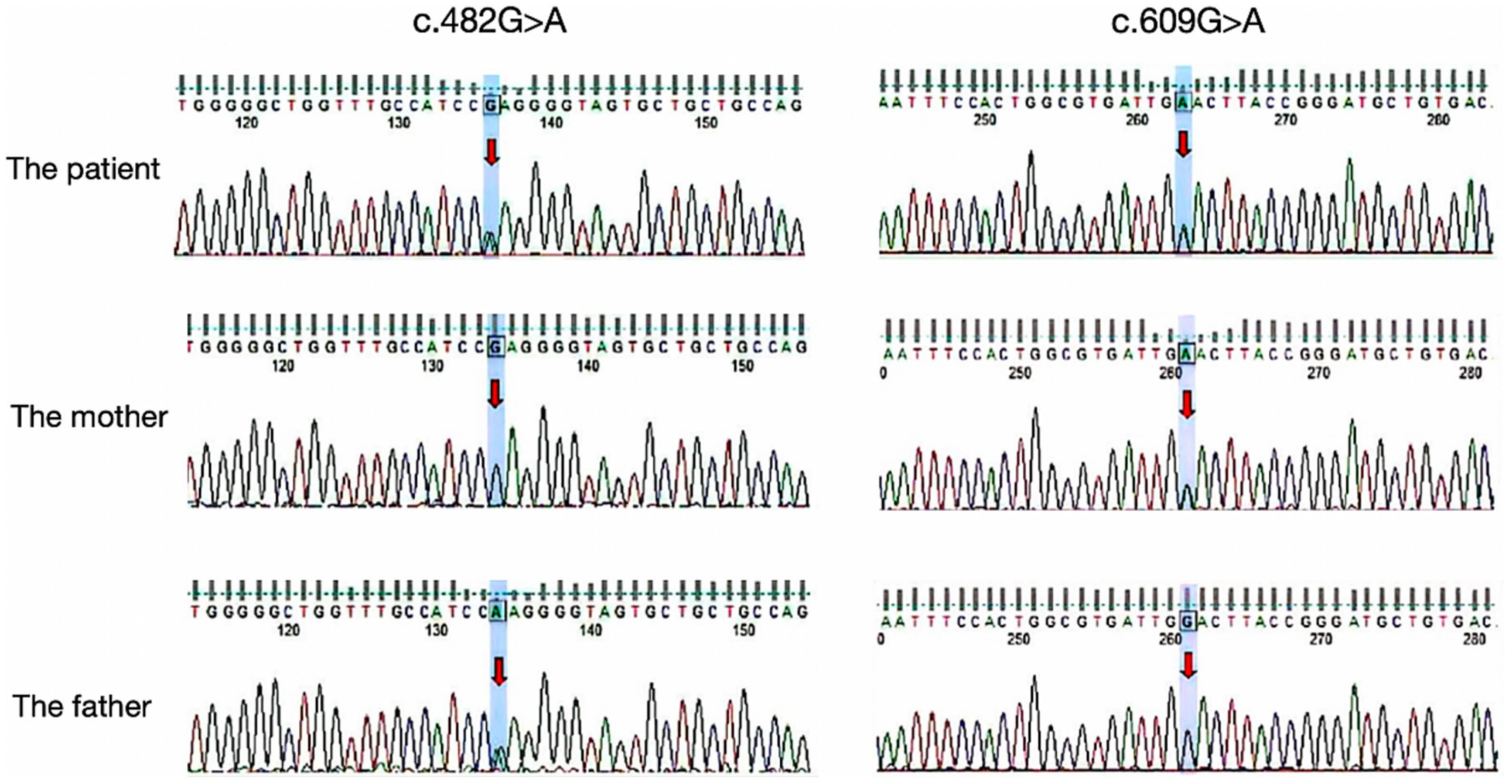
Results of the MMACHC gene test. Gene variant in c.482G > A and c.609G > A (red arrows). c.609G > A (p.Trp203*) was a nonsense mutation from his mother and c.482G > A (p.Arg161Gln) is a missense mutation derived from the patient’s father.

Auxiliary examinations, including blood routine, biochemistry, thyroid function, rheumatic immune-related antibodies, folic acid, vitamin B12 concentrations, cerebrospinal fluid analysis, and autoimmune encephalitis antibody examination, all yielded normal results. Neuropsychological examination results indicated a Mini-Mental State Examination (MMSE) score of 20, a Montreal Cognitive Assessment (MoCA) score of 18, an International Cooperative Ataxia Rating Scale (ICARS) score of 41, and a Barthel Index (BI) score of 55. MRI revealed cortical atrophy and symmetric hyperintensities in the bilateral lateral cerebellar hemispheres, primarily observed on DWI (Figure 1). Liquid chromatography tandem mass spectrometry-based blood amino acid and acylcarnitine spectroscopy demonstrated significantly elevated levels of propionylcarnitine (10.53 μmol/L, normal range: 0.30–5.00 μmol/L), the propionylcarnitine to acetylcarnitine ratio (0.50, normal range: 0.02–0.20), and the propionylcarnitine to methionine ratio (0.90, normal range: 0.02–0.30). Gas chromatography mass spectrometry-based urine organic acid analysis showed a significant increase in methylmalonic acid (76.9 nmol/L, reference value, 0.00–4.00 nmol/L). These biochemical findings were consistent with a diagnosis of methylmalonic acidemia and homocysteinemia. Whole-exome sequencing, followed by Sanger sequencing for verification, identified compound heterozygous variant in the MMACHC gene: c.609G > A (p.Trp203*), a nonsense variant inherited from the patient’s mother, and c.482G > A (p.Arg161Gln), a missense variant inherited from the patient’s father (Figure 2). According to the guidelines established by the American College of Medical Genetics and Genomics (ACMG), these variants were classified as likely pathogenic. Treatment for late-onset CblC deficiency in adulthood included intramuscular injection of methylcobalamin (0.5 mg/d), oral supplementation of vitamin B6 (30 mg/d), betaine (9 g/d), folic acid (5 mg/d), levkanitine (3 g/d), and subcutaneous injection of dalteparin (5,000 IU/d). After 1 month of treatment, the patient exhibited decreased plasma total homocysteine levels (78.1 μmol/L) and D-dimer levels (0.36), improved irritability, response times, and walking stability. Neuropsychological examination scores changed as follows: MMSE scale (24 points), MoCA Scale (20 points), ICARS Scale (10 points), and BI Scale (95 points). Additionally, the DWI imaging revealed a reversal of cerebellar signal changes. Follow up with the patient for one year. Currently, the patient’s ataxia and intelligence has further improved and he can work normally, but he is still taking medication for supplementation of methylcobalamin (1.5 mg/d), vitamin B6 (30 mg/d), betaine (9 g/d), folic acid (5 mg/d), levkanitine (3 g/d).

## 3 Discussion and conclusion

We present a case report of a 30-year-old male patient who presented with symptoms of cerebellar ataxia and cognitive impairment. Imaging studies revealed bilateral symmetric hyperintensity in the lateral cerebellar hemispheres, primarily observed on DWI. Various potential disorders were considered based on the presence of bilateral cerebellar symmetric lesions on imaging, including cerebral infarction, cerebellitis (autoimmune or infection-related), and toxic encephalopathy (metronidazole, heroin), among others (2–5). Through appropriate investigations, these differential diagnoses were ruled out. The patient’s blood tests revealed apparent abnormalities in total homocysteine levels, which have been associated with more than 100 diseases or conditions. In adults, values of 10 mmol/L or less are generally considered safe, while values of ≥11 mmol/L warrant intervention (6). Considering the impact of elevated plasma total homocysteine on the nervous system, additional investigations were conducted, including MRI of the cervicothoracic and lumbar spine and screening for organic acids in the urine. The results showed no abnormalities in the spinal MRI, while the elevation of methylmalonic acid indicated a metabolic disease. The diagnosis of late-onset cblC deficiency in adults was confirmed through genetic testing.

MMA, also known as methylmalonic aciduria, is a congenital organic acid metabolic disease with multifactorial autosomal recessive inheritance (7). Various genetic defects in methylmalonic acidemia and homocysteinemia, involving cobalamin metabolism, have been identified, including cblC, cblD, cblF, cblJ, and cblX, with cblC-deficient disease being the most prevalent (8). In 2006, the protein encoded by MMACHC was identified as the cause of cblC deficiency disease, with the MMACHC gene located at 1p34.1 (9). Early-onset cblC disease typically manifests during infancy or within the first year of life. However, late-onset cblC disease, which appears in adolescence or adulthood, is less common and was first reported in 1980 (10).Only around 100 cases have been reported in the literature (11), often exhibiting atypical clinical symptoms without a family history. Prompt treatment improves prognosis, but late-onset cases are highly heterogeneous and prone to misdiagnosis. On average, there is a delay of 32.1 months from the initial symptoms to diagnosis (12).

Clinical manifestations of late-onset cblC disease may include neurologic symptoms (cognitive dysfunction, myelopathy, gait abnormalities, seizures, pyramidal signs, peripheral neuropathy, and thromboembolic seizures), psychiatric symptoms (progressive cognitive deterioration, degeneration, behavioral and personality changes, social withdrawal, psychosis, confusion), hematologic manifestations, anorexia, renal failure, ocular abnormalities, dermatitis, and pancreatitis, etc. (13–15). Among the various gait abnormalities observed, the predominant manifestations include spastic paraplegia gait, sensory ataxia gait, and stride gait (16). Notably, cerebellar ataxia as a clinical or imaging manifestation is rare in cblC disease.

Neuroimaging findings in late-onset cblC disease are not extensively documented. Diffuse white matter swelling, severe leukoaraiosis of varying degrees, hydrocephalus, corpus callosum atrophy, and symmetric bilateral lesions of the basal ganglia are frequent and distinctive imaging findings in early-onset cblC disease. However, late-onset cases often exhibit cerebral atrophy and patchy lesions in the deep white matter (17–19). Cerebellar involvement is uncommon in cblC disease, and literature review reveals only seven reported cases with cerebellar symptoms or imaging changes (12, 16, 18, 20–23). We summarize the clinical symptoms or MRI differential presentation of the previously reported involving cerebellar related cases with late-onset CblC deficiency in Table 1. The age of onset of the studied cohort was approximately 20 years, and there was a balanced ratio of men to women. Only one patient had a positive family history, and the majority of patients received a diagnosis several years after the initial onset of symptoms. Notably, cognitive deterioration and psychiatric symptoms were the prominent clinical manifestations observed, with only 2 patients developing cerebellar ataxia. Imaging examinations revealed cerebellar lesions in six patients, of which two exhibited concurrent cerebral atrophy and exclusively impacted the cerebellum. One patient demonstrated bilateral cerebellar hemisphere lesions in the T2 sequence, while another patient exhibited T1 low intensity, T2, and DWI hyperintensity in both the cerebellar hemispheres and vermis, with mild enhancement of the lesions. Importantly, the maximum innovation of this article lies in imaging that we present a rare case characterized by cerebellar ataxia and cognitive impairment as the primary disturbances, bilateral lateral cerebellar hemisphere symmetric hyperintensity on DWI without change on T1WI, T2WI, T2FLAIR, and significant cortical atrophy. Among the cohort, three patients exhibited electromyography (EMG) abnormalities, primarily manifesting as sensorimotor polyneuropathy. Unfortunately, complete EMG examination was not conducted for the patients included in this report due to objective reasons, which represents a limitation of this study. Regarding genetic diagnosis, five patients carried the MMACHC gene 482G > A variant. The clinical symptoms of all seven patients improved following treatment with adenosylcobalamin, hydrocobalamin, cyanocobalamin, folic acid, and betaine. Notably, hydrocobalamin drugs, although characterized by long half-lives and easy absorption, are not readily available in the domestic market. In this particular case, the patient received intramuscular injections of methylcobalamin, which led to enhanced clinical symptoms and improved imaging findings.

**Table 1:** The clinical symptoms or MRI differential presentation involving cerebellar related cases with late-onset CblC deficiency.

Patient no. [Reference]	Diagnose age	Sex	Clinical symptoms	MRI results	Urine MMA (μM/L)	Serum Hcy (μM/L)	Gene mutations	Gene sites	Outcome
1 (our patient)	30	Male	Cognitive impairment, cerebellar ataxia	Cortical atrophy and symmetric hyperintensities in the bilateral lateral cerebellar hemispheres just on DWI	76.9	296.90	MMACHC	c.609G > A c.482G > A	Improved
2 (12)	14	Female	Cognitive impairment	Hyperintensity in the bilateral cerebellar on T2WI	185.8	110	MMACHC	c.482G > A c.445-446del	Improved
3 (16)	21	Female	Spastic paraplegic gait, lower limb muscle strength decreased	Hyperintensity in the bilateral cerebellar, impaired myelination and periventricular hyperintensity on T2WI	332.9	154.5	MMACHC	c.482G > A c.658-660del	Improved
4 (18)	10	Male	Ataxia gait	Cerebellum, cervical and thoracic cord atrophy	102	89.2	MMACHC	c.217C > T c.615C > A	Improved
5 (20)	29	Female	Cognitive impairment	Abnormal signals in the vermis and in the bilateral cerebellar hemispheres with low signal on T1-weighted image, high signal on T2-weighted image and high signal on DWI sequence with Gd enhancement	306.4	178.41	MMACHC	c.484G > A c.658-660del	Improved
6 (21)	15	Male	Cognitive impairment	Cortical and bilateral cerebellum atrophy	58.42	102.6	MMACHC	c.482G > A c.658-660del	Improved
7 (22)	22	Male	Dysarthria, imbalance walking, weakness of lower limbs, generalized seizure	Bilateral cerebellar on T2/ FLAIR hyperintensities, intramedullary T2 hyperintensity at C5–6 with cord atrophy at C7–D7	144	234.4	MMACHC	c.374 T > C c.394C > T	Improved
8 (23)	16	Male	Unsteady gait	MRI of the cervical spine and brain were all normal	149	185.1	MMACHC	c.567dupT c.482 G > A	Improved

**Figure 3:**
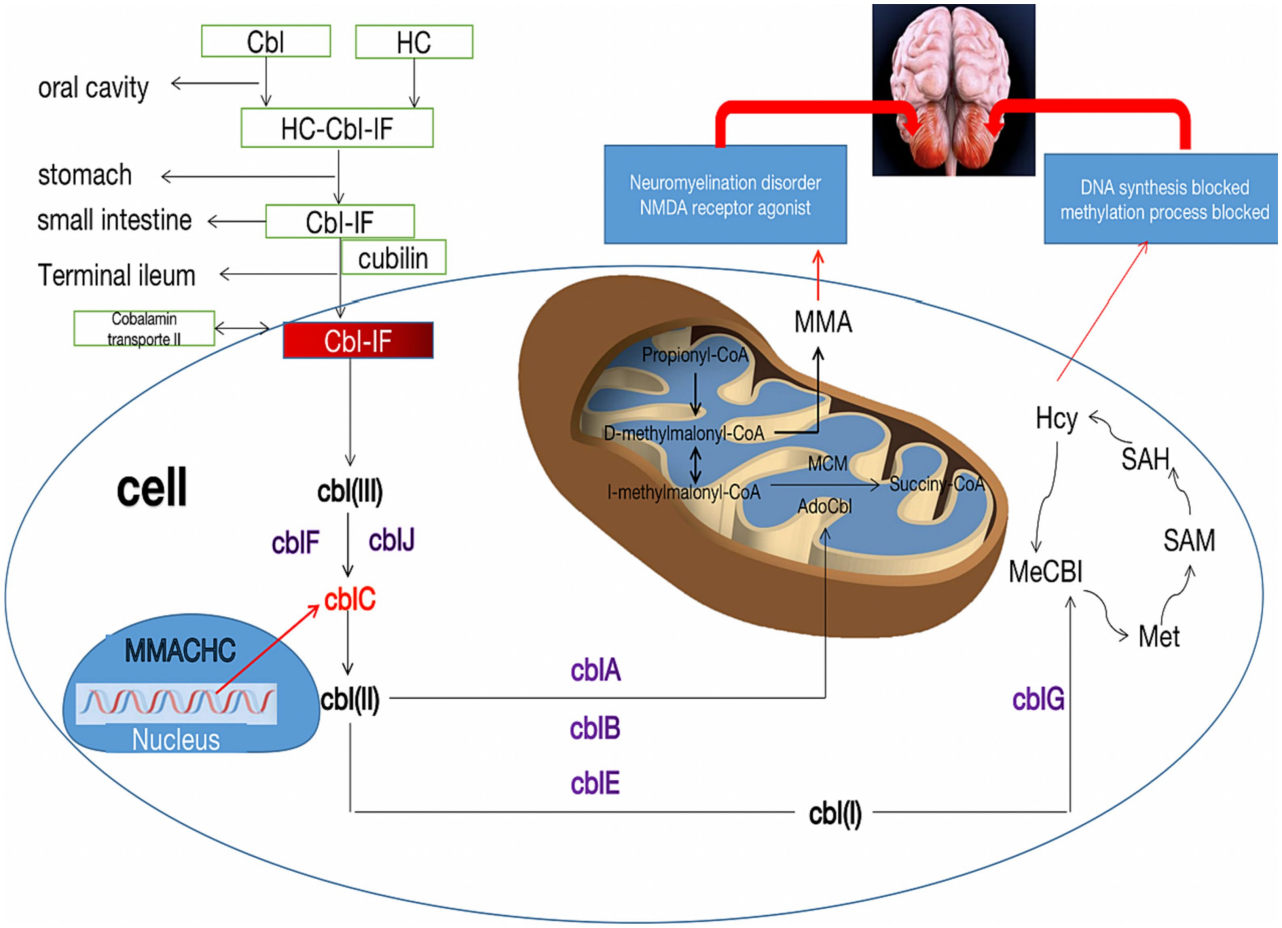
Metabolic pathways of cblC defects and its relationship with cerebellar damage. The neurotoxicity of MMA and Hcy due to the cblC defect is described for their importance in the pathophysiology of cerebellar damage. Cbl, cobalamin; HC, haptocorrin; IF, Internalfactor; cblA-G, cblJ, cbl complementation; MMA, methylmalonic acid; Hcy, homocysteine; Met, methionine; SAM, S-adenosyl-methionine; SAH, S-adenosyl-homocysteine; MMACHC, gene responsible for methylmalonic acidemia and homocysteinemia; NMDA, N-methyl-D-day aspartate.

The pathogenesis of methylmalonic acidemia involves a complex interplay of various mechanisms that contribute to its effects on the nervous system. The cause of cerebellar cytotoxic damage in this patient is unknown, but several potential causes of cerebellar involvement can be considered. Firstly, the cerebellum requires lower levels of methylcobalamin due to its low activity requirements for protein carboxymethylation. As a result, the supply of methylcobalamin to the cerebellar hemispheres is comparatively lower. However, prolonged cobalamin deficiency in the cerebellar hemispheres may render them more vulnerable (24). Secondly, the accumulation of homocysteine stimulates the N-methyl-D-aspartate receptor (NMDAR), leading to increased production of anti-NMDAR antibodies. While cerebellar Purkinje fibers lack NMDAR expression, the binding of anti-NMDAR antibodies to the cerebellar molecular layer and granular layer can cause cytotoxic effects (25, 26). Thirdly, the cerebellar hemisphere has a higher demand for blood, oxygen, and energy metabolism compared to the white matter. Consequently, in metabolic diseases, the cerebellar hemisphere is more susceptible to damage than the white matter. Additionally, the cerebellum is also susceptible to drug and toxin-induced damage, further increasing the likelihood of cerebellar involvement in patients with methylmalonic acidemia (27) (Figure 3). Cerebellar ataxia is commonly seen in neurological practice. There is a wide array of causes of cerebellar ataxia, including vascular, neoplastic, nutritional, metabolic, immune-mediated, infectious, toxic, paraneoplastic and hereditary. MRI is occasionally helpful in making a diagnosis. Most commonly, brain stem and/or cerebellum lesions with reduced diffusivity with ataxia suggest an acquired and acute (usually vascular, inflammatory, or toxic) cause. Despite the utility of neuroimaging in diagnosing cbIC and in identifying the loci and extent of damage, MRI examination often post-dates the acute phase marked by edema affected brain tissue. Therefore, in more cases, atrophy or abnormal signal is the most common manifestation on T2/Flair sequence, less on DWI.

To date, nearly 80 variants in the MMACHC gene have been reported, with 30 of these variants documented in China. The most frequent variant in Europe and the United States is c.271dupA, whereas the most common early-onset variant in China is c.609G > A (28). In the present case, the patient’s genetic testing revealed a compound heterozygous variant of the MMACHC genes, specifically c.482G > A and c.609G > A, which are the most commonly reported mutations in cases of late-onset cblC deficiency disease in China. Notably, among cases involving cerebellar manifestations, the c.482G > A variant is the most frequently observed. However, the precise relationship between specific gene variant sites and phenotypes remains unclear.

In conclusion, MMA is a small molecular genetic metabolic disease characterized by acute onset, recurrent courses, nonspecific physical appearance, and imaging features, with remarkable treatment effects. Our case emphasizes the importance of considering genetic metabolic diseases, especially those involving the cerebellum, in adult patients with undiagnosed neurological disorders. Methylmalonic acidemia should be considered as part of the differential diagnosis when bilateral cerebellar lesions are present. In addition, specially when preliminary investigations exclude most acquired ataxia. Coexistent clinical or subclinical neurological presentations, may support this diagnosis. Further screening for abnormal intracellular vitamin B12 metabolism, biochemical factors, or genetic mutations, may help to confirm the diagnosis and initiate timely treatment.

## Data availability statement

The datasets presented in this article are not readily available because of ethical and privacy restrictions. Requests to access the datasets should be directed to the corresponding author.

## Ethics statement

The studies involving humans were approved by the Medical Ethical Research Committee of the General Hospital of Northern Theater Command. The studies were conducted in accordance with the local legislation and institutional requirements. Written informed consent for participation in this study was provided by the participants’ legal guardians/next of kin. Written informed consent was obtained from the individual(s), and minor(s)’ legal guardian/next of kin, for the publication of any potentially identifiable images or data included in this article.

## Author contributions

MS: Writing – original draft, Writing – review & editing. YD: Writing – review & editing.
